# Recombinant Varicella-Zoster Virus Vaccines as Platforms for Expression of Foreign Antigens

**DOI:** 10.1155/2013/219439

**Published:** 2013-06-13

**Authors:** Wayne L. Gray

**Affiliations:** Department of Microbiology and Immunology, University of Arkansas for Medical Sciences, 4301 West Markham Street, Little Rock, AR 72205, USA

## Abstract

Varicella-zoster virus (VZV) vaccines induce immunity against childhood chickenpox and against shingles in older adults. The safety, efficacy, and widespread use of VZV vaccines suggest that they may also be effective as recombinant vaccines against other infectious diseases that affect the young and the elderly. The generation of recombinant VZV vaccines and their evaluation in animal models are reviewed. The potential advantages and limitations of recombinant VZV vaccines are addressed.

## 1. Introduction

 Varicella-zoster virus (VZV) vaccines provide immune protection against diseases that affect both the young and the elderly. The live, attenuated varicella vaccine (VARIVAX) immunizes children against chickenpox, a childhood disease characterized by fever and vesicular skin rash. The VZV Zostavax vaccine protects older adults against herpes zoster (shingles), a vesicular skin disease caused by VZV reactivation from latently infected neural ganglia. 

 The proven safety and effectiveness of the varicella and shingles vaccines provide support for recombinant VZV (rVZV) vaccines to induce immunity against not only VZV but also against other pathogens. The ability of VZV vaccines to safely induce long-lasting humoral and cellular immune responses provides advantages over other live, attenuated vaccine vectors and over killed and subunit vaccines. This review summarizes research to develop and evaluate VZV vectors as recombinant vaccines against other diseases. 

### 1.1. Varicella and Herpes Zoster

 Varicella is a highly contagious disease of children and adolescents [1]. The infectious agent is transmitted through aerosols by coughs and sneezes or by direct contact with rash secretions. Following a 10–14 day incubation period, fever and malaise arise along with the characteristic vesicular skin rash, which occurs on the face, torso, and extremities. Chickenpox is generally a benign disease in otherwise healthy children with symptoms usually completely resolved within two weeks of disease onset and with subsequent life-long immunity against varicella. However, complications may include varicella pneumonia, hepatitis, encephalitis, and secondary bacterial infections. Immunocompromised children, including cancer and AIDS patients, are particularly susceptible to severe, sometimes life-threatening varicella [2, 3]. Adults who escape VZV exposure during childhood have more severe varicella symptoms and complications. VZV infection in pregnant women during the first 28 weeks of gestation may lead to fetal infection and congenital malformations [4]. Later maternal infection may cause neonatal varicella. Prior to routine vaccination, varicella was annually responsible for 100–200 varicella-related deaths and 11,000 hospitalizations in the U.S. 

 Following resolution of the primary disease, VZV establishes life-long latent infection within neural ganglia [5]. Later in life, the virus may reactivate, travel along sensory nerves, and reinfect the skin. Herpes zoster, which occurs primarily, but not exclusively, in older adults, is characterized by unilateral pain and a vesicular rash that are limited to the dermatome innervated by a single spinal or cranial nerve [1]. Shingles affects approximately 20–30% of individuals with estimated one million cases in the U.S. each year. Postherpetic neuralgia, a common complication of zoster, is characterized by intense, sometimes chronic, pain. Immunosuppressed cancer and AIDS patients are at risk for severe herpes zoster with possible disseminated disease [3]. 

 Acyclovir and related nucleoside analogs may be effective against chickenpox and shingles, particularly if administered early in the course of infection. However, prevention against VZV disease outbreaks has been facilitated by development of VZV vaccines. 

### 1.2. VZV Vaccines

The VZV VARIVAX vaccine induces immunity and protection against chickenpox in children [1]. The vaccine, approved in the U.S. in 1995, is the first human herpesvirus vaccine licensed for clinical use. The live vaccine is derived from a clinical isolate, VZV Oka, and was attenuated by passage in guinea pig embryo and human WI38 cells, although the molecular basis of attenuation remains unknown. An initial immunization is recommended for children 12–15 months of age followed by a booster vaccination at ages 4 to 6. Administered by intradermal inoculation, this two-dose immunization approach reduces the incidence of varicella in children by 90–95%. The varicella vaccine is now recommended as a routine childhood vaccination in the U.S. with over 80% of school-age children immunized each year. Vaccination is also beneficial for susceptible adults, who are at risk for severe varicella. The vaccine is well tolerated with only minor side effects, including soreness at the inoculation site, fever, and mild rash. The VZV vaccine is even safe and effective for some cancer patients and for some immunosuppressed patients, including HIV-infected children with depression of CD4+ T cells [6]. The vaccine is not recommended for pregnant females. The VZV Oka vaccine virus is capable of establishing latent infection in neural ganglia and may reactivate to cause herpes zoster, although the incidence appears to be significantly less than that caused by wild-type VZV [7, 8]. 

 The shingles (Zostavax) vaccine is also composed of live, attenuated VZV Oka but is administered at a >10-fold higher viral dose than the varicella vaccine [9]. The vaccine, administered by subcutaneous injection, reduces the incidence of shingles and postherpetic neuralgia overall by 50% and 67%, respectively, and lasts at least for 5 years [10]. The U.S. Food and Drug Administration (FDA) approved the shingles vaccine in 2006 for individuals aged 50 and older. The Centers for Disease Control (CDC) recommend that adults aged 60 and older receive this vaccine. Vaccine efficacy decreases with advancing age (64% in persons 60–69 years, 41% in persons 70–79, and 18% in persons over 80) [11]. Only minor side effects are observed, including possible irritation at the site of inoculation and headache. The shingles vaccine is not approved for immunocompromised individuals, although it has been shown to be safe and effective in some cancer patients [12]. 

## 2. The VZV Oka Vaccine as a Potential Recombinant Vaccine Vector

 The varicella and shingles VZV vaccines are candidates as vehicles for delivery of foreign antigens and immunization against other infectious agents. VZV vaccines are highly immunogenic inducing long-lasting neutralizing antibody and cellular immune responses. In addition to their proven safety, efficacy, and widespread use, the vaccines offer advantages as a vector for the expression of heterologous antigens

1. The size of the VZV genome (125 kb) allows stable insertion of multiple heterologous genes into specific loci without effecting viral replication [28].
2. VZV replicates in the cell nucleus, so foreign genes may be properly spliced, and viral antigens are expressed, processed, and presented in infected cells as they are in natural infection [25].
3. The replication competent virus amplifies foreign antigen expression—a potential advantage over replication defective vectors.
4. A protective Th1 type T-cell immune response against VZV and the foreign antigen is induced [26].
5. VZV DNA does not contain genetic elements associated with oncogenesis.
6. The VZV host range is limited to man, limiting uncontrolled environmental spread.
7. Periodic subclinical reactivation of VZV from latency may provide restimulation of immune responses to VZV and foreign antigens as discussed below [58].

A recombinant varicella or shingles vaccine could be ideally suited for prevention of other diseases that affect children or the elderly, respectively. An rVZV vaccine offers greater safety as a general population vaccine compared to other live viral vectors, such as vaccinia virus, and the varicella vaccine is the only live viral vaccine approved for certain groups of cancer and otherwise immunosuppressed patients, including children with HIV infection [13]. 

## 3. Genetic Approaches for Generation of rVZV

 Initial approaches to insert foreign genes into the VZV genome involved cotransfection of susceptible human melanoma cells (MeWo) with genomic VZV DNA and a foreign gene flanked by homologous VZV DNA sequences. Genetic recombination yielded rVZV plaques which were detected by immunohistochemistry using antibody to the foreign gene product [14]. rVZV was plaque purified upon serial propagation. While successful, this approach was laborious, due to the cell-associated nature of VZV, making it difficult to clonally isolate rVZV. 

**Figure 1:**
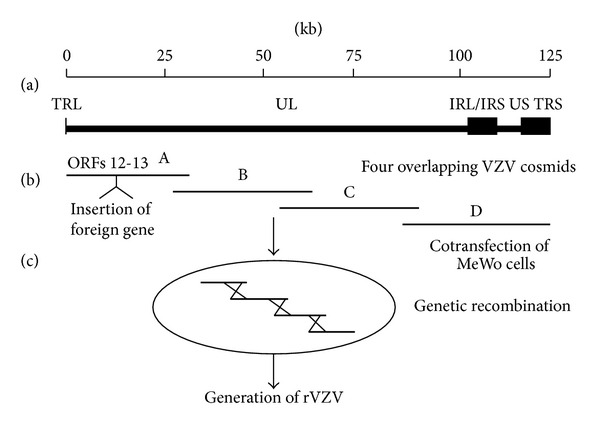
Cosmid approach to generate rVZV. (a) The 125 kb VZV genome consists of a unique long (UL) component flanked by internal and terminal inverted repeat sequences (IRL and TRL) covalently linked to a unique short (US) component bracketed by internal and terminal inverted repeats (IRS and TRS) [17]. (b) Four cosmids (30–45 kb), representing the entire VZV genome, are transfected into MeWo cells yields infectious virus. In this example, a foreign gene with a promoter is inserted into cosmid A, within the intergenic region between VZV ORFs 12 and 13.

 Development of a cosmid-based system for manipulation of the VZV genome was an important advance in VZV genetics [15]. The 125 kb VZV genome was cloned into four overlapping 30–45 kb cosmids which are cotransfected into MeWo cells. Recombination of the homologous overlapping DNA sequences yields infectious rVZV and viral plaques within ten days (Figure 1). This approach facilitates creation of rVZV mutants by site-specific mutagenesis or insertion of foreign genes within individual VZV cosmids by RecA-assisted restriction endonuclease (RARE) cleavage [16], followed by transfection of all four VZV cosmids into MeWo cells. The effectiveness of this system is limited by the requirement for efficient transfection of all four of the cosmids into the same cell and multiple recombination events.

**Figure 2:**
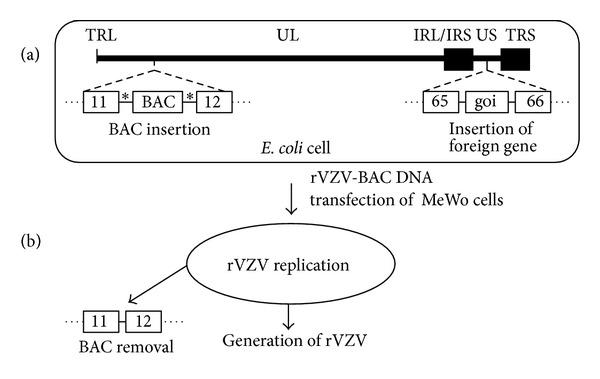
VZV-BAC approach to generate rVZV. BAC sequences are inserted into the VZV genome within the intergenic region between ORFs 11 and 12 and maintained within *E. coli*. A foreign gene of interest (goi) with promoter is inserted between ORFs 65 and 66. rVZV-BAC DNA is transfected into MeWo cells to generate infectious rVZV. BAC sequences flanked by *lox P *sites (∗) may be removed from the VZV genome by *Cre* recombination.

 An improvement over the cosmid system was insertion of the entire VZV genome into a bacterial artificial chromosome (BAC) which allowed stable maintenance of the VZV genome within *E. coli *(Figure 2) [18–20]. Infectious VZV can be reconstituted upon transfection of rVZV-BAC DNA into MeWo cells. The BAC sequences have been inserted within the intergenic region of VZV open reading frames (ORFs) 11 and 12 [18, 19] or between ORFs 65 and 66 [20] and are generally flanked by *loxP* sites, permitting removal of the bacterial vector sequences from the VZV genome by *Cre *recombination. A recent advance permits markerless, self-excision of the BAC sequences upon reconstitution in MeWo cells [20]. Development of a VZV-BAC provides an efficient approach for introduction of site-specific mutations and insertions, including foreign genes, into the VZV genome. 

## 4. Insertion of Foreign Genes within the VZV Genome 

 The large size (125 kb) of VZV DNA facilitates insertion of one or multiple foreign genes within the VZV genome. VZV encodes several genes which are nonessential for *in vitro* replication and are targets for insertion of foreign genes. For example, several rVZVs have incorporated the foreign gene within the viral thymidine kinase (TK) gene [14, 21, 22]. These rVZV-TK^−^ viruses replicate as efficiently as wild-type VZV in cell culture. Generation of rVZV-TK^−^ is facilitated by selection with nucleoside analogs such as bromodeoxyuridine. However, rVZV vaccines generated by insertion of foreign genes within the viral TK gene may not be clinically useful as effective antiviral treatment for VZV infections with acyclovir and other nucleoside analogs depends on a functional TK. In other cases, the exogenous genes are inserted within the intergenic regions of VZV ORFs 12 and 13 or between ORFs 65 and 66.

 A variety of recombination methods including *RecA*+ recombination and *Red* recombination may be used to insert genes of other pathogens into specific sites within VZV cosmids or BACs taking advantage of homologous flanking VZV sequences [20, 23]. Tn7-mediated site-specific transposition has also been used to introduce a foreign gene into VZV-BAC DNA [24]. 

 Foreign genes within rVZV have been expressed from an endogenous VZV promoter, such as the TK or gE promoters [14, 21]. In these rVZVs, the foreign gene is expressed as an immediate early, early, or late gene as during natural infection. In other rVZVs, the foreign gene is expressed from the human cytomegalovirus immediate early gene promoter/enhancer (HCMV-IE) [23]. While this commonly used promoter induces strong gene expression of foreign genes, long-term constitutive expression could result in deleterious accumulation of the recombinant protein. 

## 5. Recombinant VZV Vaccines

 Several studies provide support for the VZV vaccine virus as a vector for the expression of foreign genes. Initially, an rVZV expressing the Epstein-Barr virus (EBV) membrane glycoprotein (gp350/220) was generated and plaque purified [14]. The VZV gpI (gE) promoter and initial 35 amino acids were fused in-frame with the EBV gp gene beginning at codon 21. This construct flanked by VZV TK DNA sequences was cotransfected with VZV Oka DNA into MeWo cells. Homologous recombination of TK sequences permitted insertion of the EBV gp into VZV DNA within the viral TK gene, as confirmed by Southern blot hybridization. Approximately, 10% of viral plaques expressed EBV gp antigen as detected by immunohistochemistry using EBV monoclonal antibody. The EBV antigen was glycosylated and presented on cellular plasma membranes in a manner similar to expression in EBV-infected cells [14]. The EBV gp was also demonstrated on rVZV virions by immune electron microscopy. Due to insertional inactivation of the viral TK gene, the rVZV replicated in infected MeWo cells in the presence of bromodeoxyuridine and this property can be used for selection of rVZV.

 An rVZV Oka expressing the hepatitis B surface antigen (HBs) was generated by inserting the HBs gene into the viral TK ORF [21, 25]. The HBs gene was expressed from the VZV TK promoter, initiated at the VZV TK ATG initiation codon, and followed by codons for 25 amino acids of the HBs pre-S2 and the entire HBs protein. The construct was cotransfected with VZV Oka DNA into MRC-5 (human lung) cells. rVZV-HBs virus was detected by immunofluorescence using monoclonal antibody to HBs and was plaque purified. The HBs was synthesized as 26K and 30K proteins within infected cells. The HBs was glycosylated with N- and O-linked glycans and secreted as 30K and 35K proteins in the culture supernatant [25]. Immunization of guinea pigs with rVZV-HBs by subcutaneous inoculation induced antibody titers to HBs comparable to that induced by recombinant HBs generated in yeast. In addition, rVZV-HBs induced Th1-type cell-mediated immune responses including delayed-type hypersensitivity [26].

 The herpes simplex type 2 (HSV-2) glycoprotein D (gD) was inserted into the unique short region of the VZV Oka genome within the intergenic regions between ORFs 65 and 66 using the VZV cosmid genetic system [27]. HSV-2 gD expression was driven more efficiently from the endogenous HSV-2 gD promoter than the VZV gE promoter. The HSV-2 gD was expressed within the cell cytoplasm and on the surface of rVZV-HSV2gD infected cells as indicated by immunofluorescence and immunoblot analysis using HSV-2 gD-specific antibody. In addition, immune electron microscopy detected HSV-2 gD on the envelope of rVZV-HSV2gD virions. The HSV-2 gD expression did not affect viral replication as the VZV-HSV2gD replicated as efficiently as wild-type VZV Oka in cell culture. The VZV genome does not encode a gD homolog which may be related to its species specificity for a limited number of human cell lines. However, addition of the HSV-2 gD did not enhance the permissiveness VZV for cells which it does not efficiently replicate. Immunization of guinea pigs with rVZV-HSV2gD induced neutralizing antibodies against HSV-2 and significantly reduced disease severity, including prevention of hindlimb paralysis and mortality following intravaginal HSV-2 challenge. In a subsequent study, an rVZV Oka expressing both the HSV-2 gD and gB was constructed [28]. The HSV-2 gD was inserted within the VZV ORF 13 and expressed from the natural HSV-2 gD promoter, while the HSV-2 gB was inserted within the ORFs 65-66 intergenic region and driven by the SV40 early promoter. Immunization of guinea pigs by subcutaneous inoculation induced humoral and cellular immune responses against HSV-2 gB and gD, including neutralizing antibodies, and reduced viral shedding (>100 fold), disease severity, and mortality following intravaginal HSV-2 challenge. However, the rVZV-HSV2gD and rVZV-HSV2gBgD vaccines did not prevent HSV-2 latency and reactivation. 

 An rVZV vaccine expressing the HIV-1 env gene was constructed by insertion of the HIV-1 env gene encoding amino acids 296–463 fused to HBs antigen within the VZV TK gene [22]. The rVZV-HIVenv was selected by resistance to sorivudine, a nucleoside analog, and immunofluorescence employing an HIVenv monoclonal antibody. rVZV-HIVenv immunization induced humoral and cellular immune responses against the HIV gp120 antigen in vaccinated guinea pigs. 

 The mumps virus (MV) hemagglutinin-neuraminidase (HN) gene was inserted within the VZV Oka ORF 13 using the VZV-BAC system [23]. The HN was efficiently expressed in the cytoplasm and on the surface of rVZV-MVHN infected cells but not within the envelope of purified virions. The rVZV-MVHN replicated as efficiently as wild-type VZV, and cell tropism was not altered. Immunization of guinea pigs induced neutralizing antibodies against VZV and MV. A subsequent study showed that MV fusion (F) protein could be inserted into the rVZV genome, and the F protein was efficiently expressed in rVZV-MVF infected cells [24]. 

 The ability of an rVZV vaccine to induce immunity against simian immunodeficiency virus (SIV) was investigated in a nonhuman primate model [29]. The SIV env gene expressed from the HCMV IE promoter was inserted into the VZV genome between ORFs 65 and 66. Rhesus monkeys, immunized with rVZV-SIVenv by intranasal, intratracheal, and intramuscular inoculation, generated antibodies to VZV and SIV env as indicated by ELISA and immunoprecipitation. However, SIV-specific cytotoxic T lymphocyte or CD8+ T-cell responses were not detected. Following challenge with pathogenic SIVsmE660, rVZV-SIV immunized animals exhibited increased SIV titers, rapid CD4 T-cell proliferation, and enhanced progression to simian AIDS, compared to control monkeys immunized with wild-type VZV. The authors speculated that the limited ability of VZV to replicate in tissues of rhesus monkeys led to inefficient induction of CD8+ T-cell responses and that the CD4 T-cell proliferation resulted in higher SIV replication and enhanced disease progression. Thus, nonhuman primates may not be the optimal animal model to evaluate rVZV-SIV vaccines. The problem can be avoided by using the simian varicella model to evaluate potential recombinant varicella vaccines as described below. 

 rVZV expressing foreign genes can also be employed to investigate the molecular basis of viral pathogenesis. The VZV cosmid system was used to generate rVZV Oka expressing a fusion protein consisting of the immediate early gene ORF 63 (IE63) and its duplicate gene ORF 70 fused in-frame to click beetle luciferase genes [30]. IE63 expression was detected within the cytoplasm of rVZV-63/70-*Luc-*infected melanoma cells by confocal microscopy and the virus replicated *in vitro* as efficiently as wild-type VZV. The rVZV-63/70-*Luc* retained pathogenicity in the SCIDhu mouse model of VZV pathogenesis. *In vivo* IE63 expression over the course of at least 21 days was measured in rVZV-63/70-*Luc*-infected human skin and dorsal root ganglia xenografts by whole animal imaging. 

## 6. Simian Varicella, a Model to Evaluate Recombinant Varicella Vaccines

 Studies on VZV pathogenesis, antiviral therapy, and vaccines are hampered because VZV inoculation of laboratory animals, ranging from mice to monkeys, fails to induce a varicella-like disease. However, a natural disease closely resembling human varicella occurs in nonhuman primates with symptoms characterized by fever and vesicular skin rash [31]. Simian varicella epizootics occur sporadically in facilities housing Old World monkeys [32]. In some epizootics, the clinical symptoms are mild, similar to the rather benign varicella seen normally in children. In others, a more severe, disseminated varicella disease is observed with symptoms similar to those seen in some adults and immunosuppressed patients. 

 The causative agent, simian varicella virus (SVV), is a primate herpesvirus with properties resembling human VZV. The SVV and VZV genomes are similar in size and structure, share 70–75% DNA homology, and are colinear in gene organization [33–35]. SVV and VZV are antigenically related as indicated by the ability of VZV immunization to confer immune protection against acute simian varicella in monkeys following SVV challenge [36, 37]. Like VZV, SVV establishes latent infection in neural ganglia and may reactivate to cause secondary disease similar to herpes zoster [38, 39]. 

 Based upon the genetic and antigenic relatedness of SVV and VZV and the clinical similarities between simian and human varicella, SVV infection of primates is a useful animal model for studying viral pathogenesis and for evaluating the effectiveness of antiviral agents and vaccines [31, 40, 41]. 

 The SVV model offers an approach to evaluate recombinant varicella vaccines. The initial recombinant SVV (rSVV) was generated by insertion of the green fluorescent protein (GFP) gene expressed from the Rous sarcoma virus (RSV) promoter into the intergenic region between SVV ORFs 65 and 66 by homologous recombination [42]. Insertion of the GFP gene into this region of the SVV genome did not inhibit viral growth in cell culture. The rSVV-GFP was pathogenic in African green monkeys inducing necrotizing pneumonitis upon intratracheal inoculation. GFP expression was detected within infected cells derived from lung tissues on day 10 postinfection. 

 An SVV cosmid genetic system was employed to generate an rSVV expressing SIV antigens. The SIV gag and env genes were inserted within the SVV glycoprotein C (ORF 14) gene, and SIV antigen expression in infected Vero cells was confirmed by immunofluorescence and immunoblot analysis using SIV monoclonal antibodies [43]. The rSVV-SIVenv and rSVV-SIVgag replicated as efficiently in infected Vero cells as wild-type SVV. The rSVV-SIVenv and rSVV-SIVgag were initially evaluated by coinfection in infected African green monkeys inoculated by intratracheal and subcutaneous inoculation. The viruses were attenuated in the monkeys as indicated by reduced skin rash and viremia compared to wild-type SVV infection [43, 44]. The attenuation is likely due to the insertional inactivation of the SVV gC, which has been associated with VZV and herpesvirus virulence [45–47]. The rSVV-SIVgag and SVV-SIVenv vaccines induced humoral and cellular immune responses to the SIV antigens as revealed by ELISA and ELISPOT analyses, respectively. Each of the viruses also established viral latency as indicated by detection of rSVV DNA in neural ganglia by PCR analysis.

 A subsequent study evaluated the ability of the rSVV-SIVenv and rSVV-SIVgag vaccines to immunize rhesus macaque monkeys against SIV infection [44]. The rSVVgag/env immunization induced antibody and cellular immune responses against SIV. Six months following the rSVV immunization, the monkeys were challenged with pathogenic SIV strain SIVmac251-CX-1 by intravenous inoculation. The rSVV-SIVgag/env immunization reduced SIV plasma viral loads by 100-fold in rSVV-SIVgag/env immunized monkeys compared to monkeys immunized with a negative control rSVV. Increased CD4+ T-cell proliferation and SIV-specific polyfunctional cytokine responses correlated to the reduced viremia in rSVV-SIVgag/env immunized monkeys [48]. The results of this study suggest that an rVZV-HIV vaccine could be an effective approach for AIDS vaccination. 

 An rVZV vaccine expressing respiratory syncytial virus (RSV) antigens could be effective for immunization of children or older adults who are most susceptible to RSV-induced respiratory disease. To evaluate a recombinant varicella vaccine, rSVV expressing the RSV glycoprotein G and the M2 matrix protein were constructed employing the SVV cosmid system [49]. Immunization of rhesus monkeys with the rSVV-RSVG and M2 vaccines induced neutralizing antibody responses against RSV. 

 The 125 kb SVV genome has recently been cloned into a BAC and stably maintained in *E. coli* [50]. The SVV BAC has been used to insert site-specific mutations within the SVV genome using *Red-*mediated recombination. The SVV BAC genetic system will facilitate the generation of rSVV expressing foreign genes and evaluation of candidate recombinant vaccines.

## 7. Influence of Preexisting VZV Immunity and Viral Latency on rVZV Vaccines

 A recombinant varicella vaccine may be effective for VZV seronegative children. However, older children and adults are generally VZV seropositive either by natural VZV infection or by prior VZV vaccination. Preexisting antibodies to VZV could potentially hamper the effectiveness of an rVZV vaccine, possibly by neutralization of the vaccine virus. Indeed, humoral and cellular immune responses to a model antigen expressed by a recombinant HSV-1 were reduced in HSV-1 seropositive mice [51]. However, effective immunization under this constraint may be feasible. Preexisting immunity to the vector may not hamper immune responses to the recombinant antigen when the vaccine is administered by a route different from that of natural infection. For example, subcutaneous or mucosal immunization is not inhibited by prior systemic infection [52, 53]. In addition, the immunogenicity of some recombinant vectors, such as cytomegalovirus, measles, and Sindbis virus, are not influenced by preexisting immunity to the vector [54–56]. The influence of preexisting immunity on the immunogenicity of rVZV vaccines has not been investigated but is an important factor to be addressed to assess vaccine safety and effectiveness. 

 The VZV vaccine virus, like wild-type VZV, can establish latent infection in neural ganglia and has the potential to reactivate to cause herpes zoster [7, 57]. However, the incidence of reactivation disease for the VZV vaccine virus appears to be significantly less than that caused by wild-type VZV [7]. VZV may undergo sporadic subclinical reactivation resulting in periodic restimulation of VZV immune responses and life-long immunity against VZV [58]. Subclinical reactivation of an rVZV vaccine virus could be an advantage, providing sporadic immune boosts against both the VZV antigens as well as the recombinant antigen. 

## 8. Conclusions

 Viral vectors offer novel opportunities as vaccine platforms, particularly against diseases for which traditional vaccine approaches have not been effective. Recombinant viral vaccines against several human diseases are currently in clinical trials [59]. Most of these recombinant vaccines utilize adenovirus or poxvirus as viral vectors. The attenuated, replication-competent VZV Oka vaccine, with widespread use, an established safety profile, and an ability to induce long-lasting antibody and T-cell-mediated immunity provides an opportunity to develop effective recombinant vaccines. rVZV vaccines could be utilized as a primary vaccine or as part of a heterologous prime-boost strategy coupled with a DNA vaccine or adenovirus/poxvirus combination. rVZV varicella and shingles vaccines offer the flexibility to develop immunization strategies against diseases that affect children and the elderly, respectively.
